# ctDNA Detection Based on DNA Clutch Probes and Strand Exchange Mechanism

**DOI:** 10.3389/fchem.2018.00530

**Published:** 2018-10-31

**Authors:** Huan Chang, Yiyi Zhang, Fan Yang, Changtao Wang, Haifeng Dong

**Affiliations:** ^1^Beijing Advanced Innovation Center for Food Nutrition and Human Health, Beijing Technology and Business University (BTBU), Beijing, China; ^2^Beijing Key Laboratory for Bioengineering and Sensing Technology, Research Center for Bioengineering and Sensing Technology, School of Chemistry and Bioengineering, University of Science and Technology Beijing, Beijing, China

**Keywords:** ctDNA, clutch probes, discrimination probes, strand displacement reaction, selectivity

## Abstract

Circulating tumor DNA (ctDNA), originating directly from the tumor or circulating tumor cells, may reflect the entire tumor genom and has gained considerable attention for its potential clinical diagnosis and prognosis throughout the treatment regimen. However, the reliable and robust ctDNA detection remains a key challenge. Here, this work designs a pair of DNA clutch separation probes and an ideal discrimination probes based on toehold-mediated strand displacement reaction (TSDR) to specifically recognize ctDNA. First, the ctDNAs were denatured to form ssDNAs, the pair of DNA clutch separation probes [one of which modified onto the magnetic nanoparticles (MNPs)] are used to recognize and hybridize with the complemental chains and prevent reassociation of denatured ssDNAs. The complemental chains are removed in magnetic field and left the wild and mutant ssDNA chains in the supernatant. Then, the TSDR specificity recognizes the target mutant sequence to ensure only the mutated strands to be detection. The proposed assay exhibited good sensitivity and selectivity without any signal amplification. The proposed assay displayed a linear range from 2 to100 nM with a limit of detection (LOD) of 0.85 nM, and it was useful for ctDNA biomedical analysis and clinic theranostic.

## Introduction

In recent years, many advanced analytical methods have been established to quantify DNAs and RNAs (Schwarzenbach et al., 2011; Si et al., 2014; Li et al., 2015; Wang et al., 2018), among them, liquid biopsy has increasingly attracted intense attention due to its rapid, cost-effective and non-invasive properties. Circulating tumor DNA (ctDNA), originating directly from the tumor or circulating tumor cells, is an effective diagnostic biomarker existing as a single or double strand in peripheral blood (Zou et al., 2017). It is potential surrogate for the entire tumor genome and has gained considerable attention for cancer diagnosis and prognosis (Das et al., 2016). It is reported that ctDNA levels could reflect the tumor burden, and patients with advanced tumors showed higher concentrations of ctDNA in plasma than that with earlier stage. For example, it was demonstrated that the level of ctDNA was related to the whole body tumor load and ctDNA decreased after complete surgery (Diehl et al., 2008). It was reported that ctDNA was detected in 82 and 47% for patients with stage IV and I disease, respectively (Bettegowda et al., 2014). Additionally, the half-life of ctDNA is very short, and it was promising for monitor the status of the tumor (Bettegowda et al., 2014; Diaz and Bardelli, 2014). Therefore, the detection of specific cancer-related sequences in ctDNA is very important in clinical application.

The detection of the ctDNA is difficult since the special double helix structure and single stranded DNAs (ssDNAs) formed during the annealing process (Noh et al., 2015), which require effective methods to prevent ssDNA from re-annealing for selectively detection of ctDNA (Das et al., 2016). Currently, DNA sequencing and polymerase chain reaction (PCR) are conventional methods for monitoring ctDNA in the blood (Murtaza et al., 2013; Bettegowda et al., 2014; Newman et al., 2014). However, both of them suffer some deficiencies including the biological environment interference, time-consuming, and cost-ineffective (Dewey et al., 2012; Wang et al., 2014). Therefore, the robust, sensitive, and selective detection of ctDNA are still urgently needed.

Two significant prerequisites for ctDNA detection are separation probes effectively preventing ssDNA from re-annealing and discrimination probes specifically recognizing the target ssDNA. The hybridization specificity based on based pairing almost requires the string hybridization condition or chemically complex nucleotide modifications. Toehold-mediated strand displacement reaction (TSDR) is widely applied in the dynamic DNA assembly for biomedical application by regulating the reaction rate (Yurke and Mills, 2003; Zhang and Winfree, 2009). Rationally designed hybridization probes based on strand exchange mechanisms or strand displacement reaction is intriguing since it can effectively and robustly distinguish single-base mutation in the gene fragment in various conditions (Xiao et al., 2005; Wang and Zhou, 2008).

**Figure 1:**
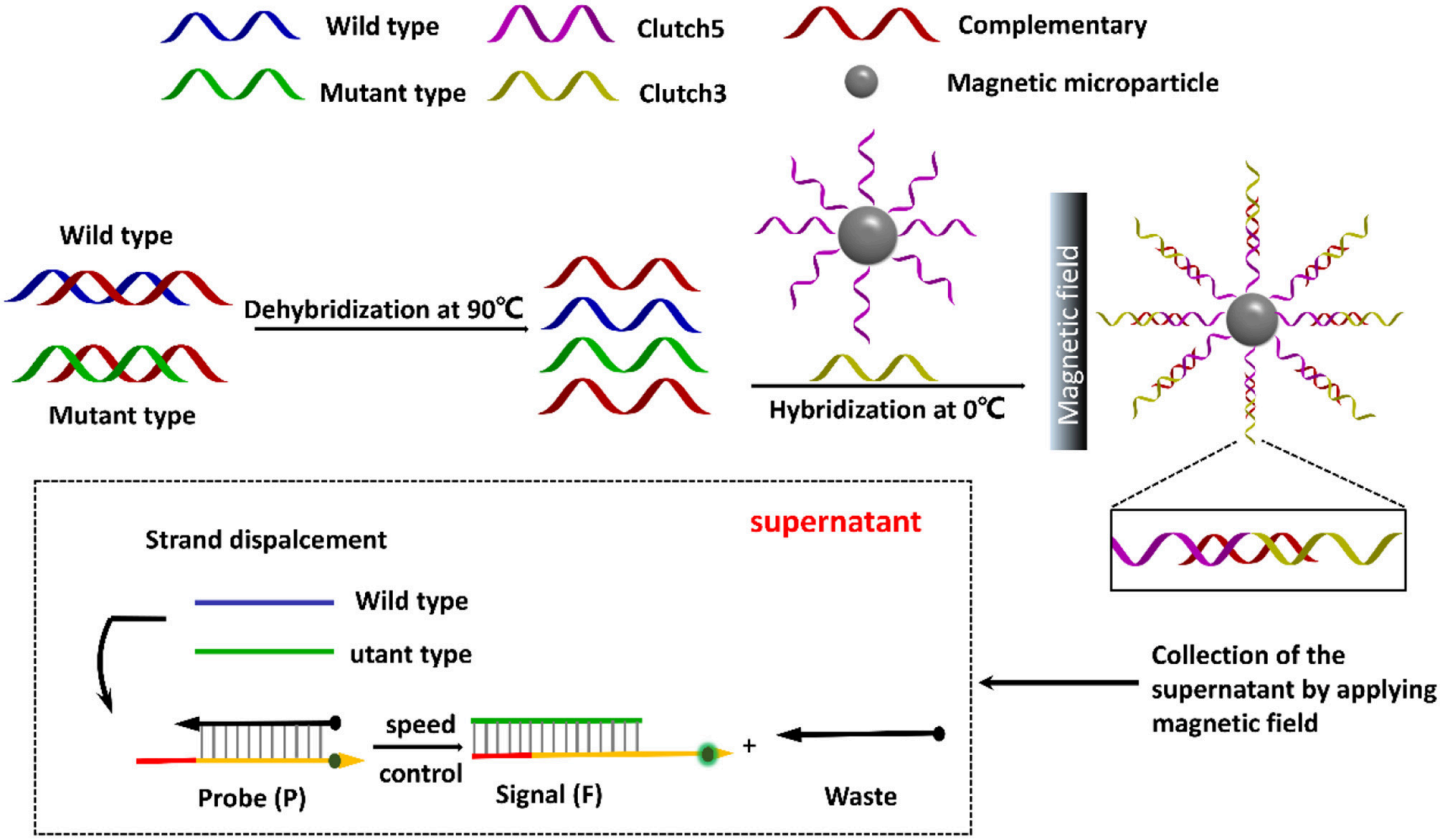
Schematic illustration of direct detection of ctDNA based on DNA clutch separation probes and TSDR reaction.

Herein, this work designs a pair of DNA clutch separation probes and an ideal discrimination probes based on TSDR to specifically recognize mutant ctDNA (Figure 1). Under annealing, the pair of DNA clutch separation probes clutch 3 and clutch 5 could recognize and hybridize with the complementary chains (red color) and prevent reassociation of denatured ssDNAs. The complementary chains were then removed in magnetic field and the wild and left the mutant ssDNA chains in the supernatant. Finally, the discrimination probes based on TSDR which could specifically recognize the target mutant ssDNA sequence to produce the fluorescence signal for detection. Thus, the specific mutant ctDNA detection has been realized, displaying a good potential in the field of ctDNA biomedical application.

## Materials and methods

### Reagents and materials

1-(3-Dimethylaminopropyl)-3-ethylcarbodiimidehydrochloride (EDC), Tris (2-carboxy-ethyl), N-hydro-xysuccinimide (NHS), phosphine (TCEP), were obtained from Sigma-Aldrich (St. Louis, MO). The ultrapure water was from a Millipore water purification system (18 MΩ, Milli-Q, Millipore, USA). Magnetic nanoparticles (MNPs) (20 mg/mL) modified with carboxyl (-COOH) group were obtained from Zhengzhou Innosep Biosciences Co., Ltd. (Zhengzhou, China). All of the DNA sequences were from Sangon Biological Engineering Technology & Services Co., Ltd. (Shanghai, China) and the detailed information of sequences were presented in Table 1. Transmission electron microscopy (TEM; JEM2010F, JEOL, 200 kV) was used to exam the morphology of the MNPs. Dynamic light scattering (DLS) was recorded on a Zetasizer Nano S system (Malvern Instruments, Malvern, U.K.). The UV–vis absorption measurements were performed with a UV-1800 spectrometer (Shimadzu, Kyoto, Japan). All fluorescence measurements were carried out on a F-4500 fluorescence spectrometer (Hitachi, Tokyo, Japan). The agarose gel electrophoresis images were captured with an Alliance Ld2 (Uvitec, Cambridge, U.K.).

**Table 1:** The detailed information of the DNA sequences used in the experiment.

**Oligonucleotide**	**Sequence (5^′^-3^′^)**
BRAF wild	TAGCTACAGTGAAATC
BRAF mutant	TAGCTACAGAGAAATC
Complementary sequence	GATTTCACTGTAGCTA
DNAclutch5	/NH2/(CH2)6GAAGACCTCACAGTAAAAATAGGTGATTTTGGTCTAGCTACAGT
DNA clutch3	GAAATCTCGATGGAGTGGGTCCCATCAGTTTGAAC
DNA 1	GAAATC
Probe 1	GATTTCACTGTAGCTA
DNA 2	/BHQ2/GATGTAGCTA
Probe 2	GATTTCACTGTAGCTACATC/Cy5/
DNA 3	/BHQ2/ACGATGTAGCTA
Probe 3	GATTTCACTGTAGCTACATCGT/Cy5/

*The base marked red in the table is the mutation site*.

### Characterization of carboxylated MNPs

10 mg/mL carboxylated MNPs were diluted into 0.5 mg/mL in phosphate buffer saline (PBS, pH = 5.1, 10 mM) and sufficiently sonicated for 2 h to obtain the dispersed solution, 2 μL of the sample was then dropped on a double networked supporting membrane fixed with small clips and dried using the heating plate for TEM analysis. 0.5 mg/mL MNPs was diluted with distilled water to 1 mL and added to a particle size cell for DLS measurement.

### DNA functionalization of MNPs

30 μL of MNPs (0.5 mg/mL, pH = 5.1), 30 μL MNPs probe (100 μM) and 4 mg of EDC were mixed in a centrifugal tube and reacted for 15 min. One milligram of NHS was then injected and the mixture was further reacted for 12 h in PBS solution (10 mM, pH = 7.4). Then the MNPs-DNA were washed with PBS (10 mM, pH = 7.4) for three times and stored in 120 mL of PBS (10 mM, pH = 7.4) for further use.

### Electrophoresis gel characterization

The hybridization of clutch probes and the ssDNA were performed at 0°C for 1 h, and verified by 4% agarose gel electrophoresis (100 V, 50 mA).

### Optimization of discrimination probes

The ssDNA (10 μL, 1 μM) labeled with fluorescent FAM firstly hybridized with their complementary ssDNA labeled with quenching groups (BHQ2) at 37°C for 2 h to form duplex helices discrimination probes. The target ssDNA with single nucleotide mutant (10 μL, 1 μM) or its wild type ssDNA (10 μL, 1 μM) was then added to the 100 μL of solution containing discrimination probes and incubated at desired time and temperatures. Afterwards, the fluorescence intensity was detected using a fluorescence spectrometer.

### ctDNA detection

Four microliter ctDNA with different concentrations were heated at 90°C for 2 min, and then added into solution containing 7 μL MNPs probes (20 μM) and 9 μL clutch 3 probe (20 μM) and reacted at 0°C for 60 min. The mixture was separated at a magnetic field and the supernatant was collected for further use. Ten microliter supernatant was added into 4 μL discrimination probes (10 μM) and the mixture was diluted into 200 μL and reacted at 37°C for 10 min. Afterwards, the fluorescence was scanned with a fluorescence spectrometer with excitation wavelength at 488 nm and scanning emission spectra from 505 to 700 nm.

## Results and discussion

### Characterization of MNPs-DNA

**Figure 2:**
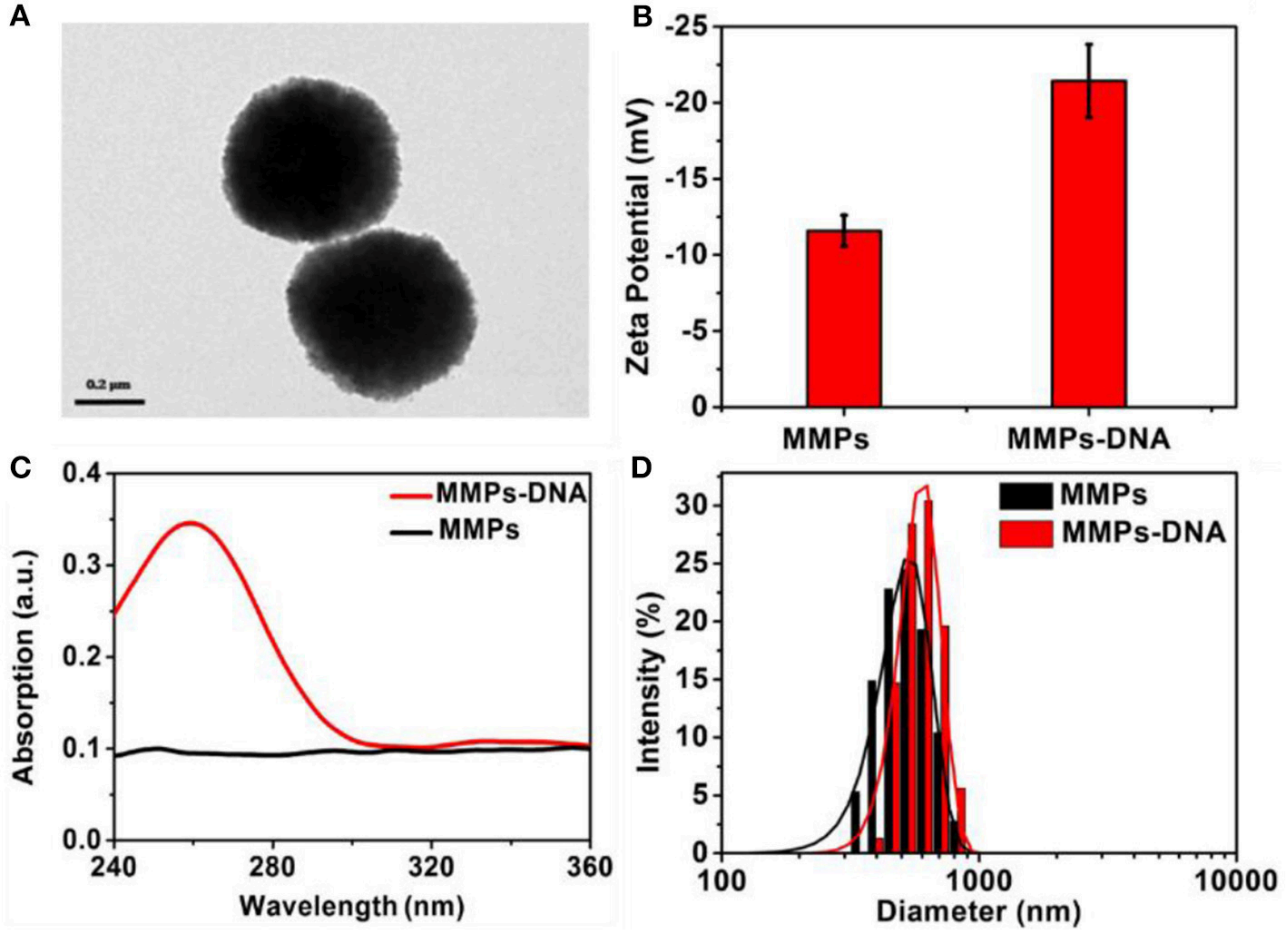
TEM image of **(A)** MNPs, Zeta potential **(B)**, UV-visible spectra **(C)**, and **(D)** DLS of MNPs and MNPs-DNA.

As shown in Figure 2A, it was obvious that the MNPs displayed uniform spherical and showed a particle size of about 500 nm. The MNP displayed a negative zeta potential of −11.8 mV, and the introduction of negatively charged DNA led to the zeta potential decreased to −22.5 mV (Figure 2B). In comparison with the UV-vis spectrum of MNPs, a strong characteristic absorption peak at 258 nm was observed in that of MNPs-DNA, revealing DNA was efficiently modified on MNPs' surface (Zhu et al., 2006; Figure 2C). The DLS analysis demonstrated the aqueous size of MNPs was approximately distributed at about 510 nm, in agreement with the TEM analysis, and it increased to 535 nm after the modification of DNA (Figure 2D). These results suggested the successful modification of DNA on the surface of MNPs. The electrophoresis experiment was employed to characterize DNA assembly. As shown in Figure S1, the lane 1–3 was the complementary sequences (16 bp), clutch probe 3 (35 bp), and clutch probe 5 (44 bp), respectively. From the lanes 4–6 analysis, it indicated the complementary sequences could hybridize with clutch probe 3 (lane 4), clutch probe 5 (lane 5), to form sandwich structure (lane 6).

### Toehold exchange probes

**Figure 3:**
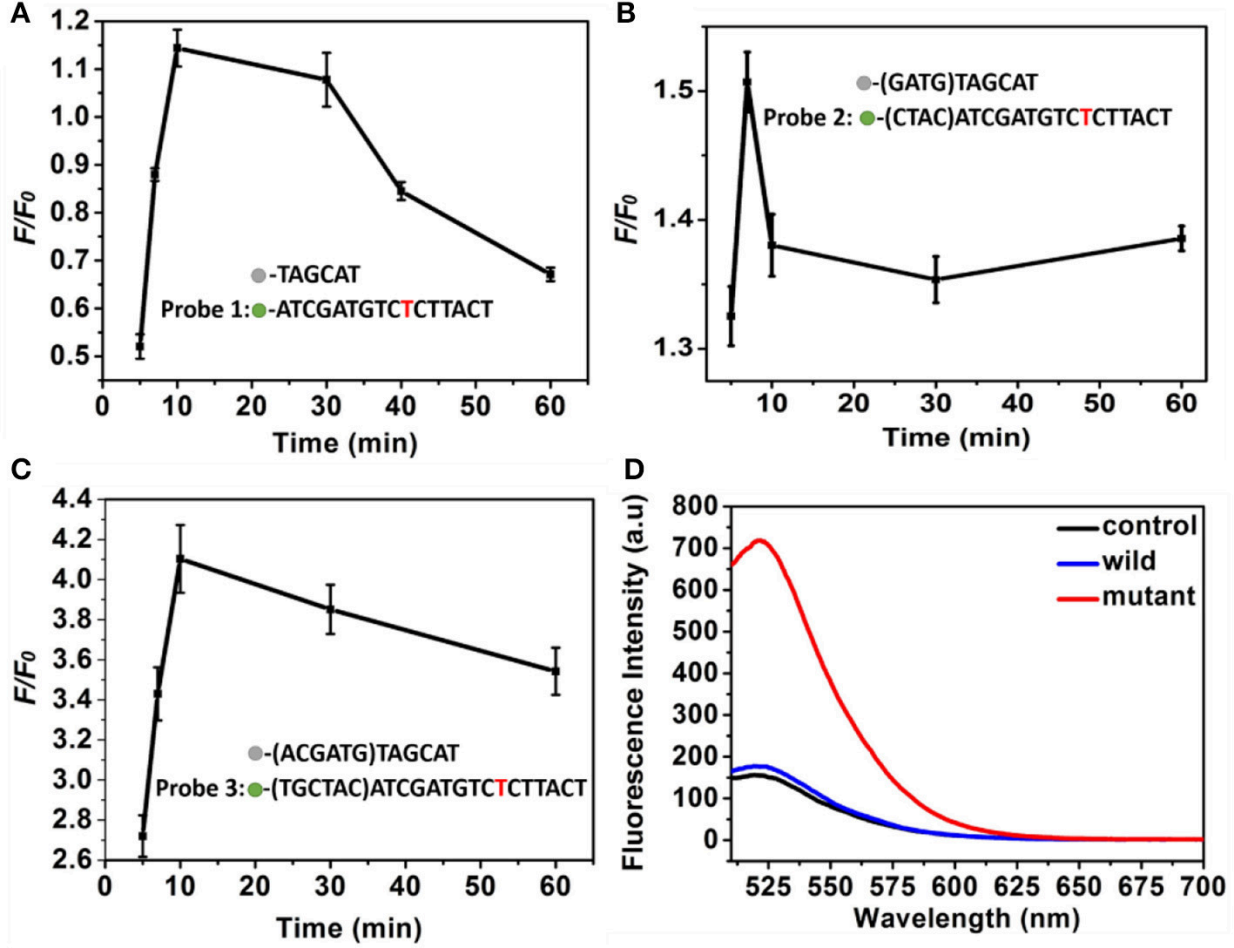
**(A–C)** The *F/F*_0_ value of **(A)** probe 1, **(B)** probe 2, and **(C)** probe 3 as a function of time. Three different discrimination probe (red is the mutant site), **(D)** Fluorescence intensity corresponding to probe 3. The concentration of the mutant type ctDNA and wild ctDNA was 100 nM.

To obtain an ideal discrimination probes with high specificity, the “toehold exchange” probes were rationally designed. The length of the cohesive ends at the 3′ end of the replacement sequence in the discrimination probes influence significantly the specificity (Chen and Seelig, 2016), which was firstly optimized. Three types of DNA probes consisting of two single sequence labeled with BHQ2 quencher and FAM fluorescent dye, respectively, were employed to investigate the optimized cohesive ends. It was found that the probe 1, probe 2, and probe 3 displayed a maximum *F/F*_0_of 1.15, 1.52, and 4.1, respectively, where the *F* and *F*_0_ was the fluorescence intensity of probes in response to the mutant-type and wild-type ctDNA (Figures 3A–C). Importantly, when the cohesive ends extended to twelve bases (probe 3), the wild-type ctDNA produced similar fluorescence intensity to the control (PBS, pH 7.4, 10 mM) group, which indicated the excellent specificity of probe (Figure 3D). Therefore, the toehold exchange probe 3 was used in subsequent experiments.

### Conditions optimization

**Figure 4:**
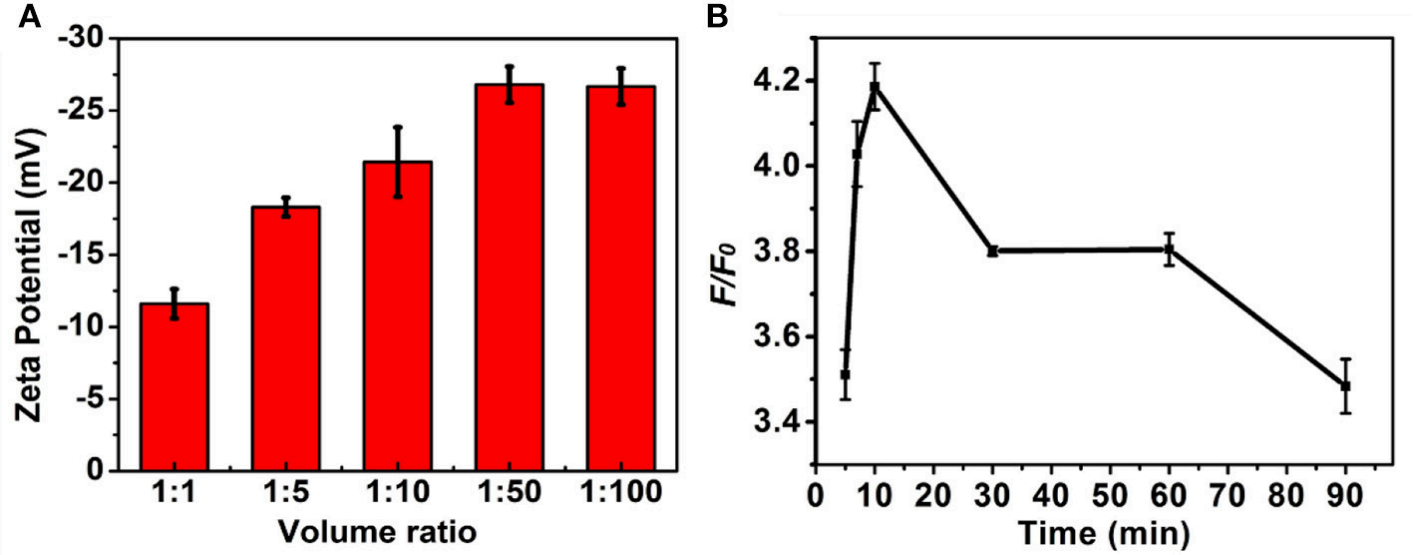
The influence of **(A)** volume ratio of MNPs (0.5 mg/mL) to DNA (1 nM) and **(B)** strand displacement reaction temperature to the *F/F*_0_. The concentration of the mutant type ctDNA and wild ctDNA was 100 nM.

The experimental conditions including the ratio of volume of MNPs and DNA in the PBS (pH 7.4, 10 mM) and strand displacement reaction time were investigated to achieve the optimal signal discriminant validity. The zeta potential analysis was used to optimize the ratio of volume of MNPs and DNA. As shown in Figure 4A, the absolute value of zeta potential gradually increased along with the increase of the ratio of volume of MNPs and DNA, and reached to maximum at the ratio of 1:50, which was selected for the followed experiments. The displacement reaction time was a significant factor in the experiment. The short reaction time might induce insufficient displacement reaction, while long reaction time could lead to decrease of the discrimination capability for wild/mutant sequence. As shown in Figure 4B, the system showed a sharply increase in the discrimination capability long with the increase of reaction time increased to 10 min, and then decreased when the reaction time further increased. Thus the 10 min was selected as the optimized reaction time.

### Sensitivity of the proposed system proposed system

**Figure 5:**
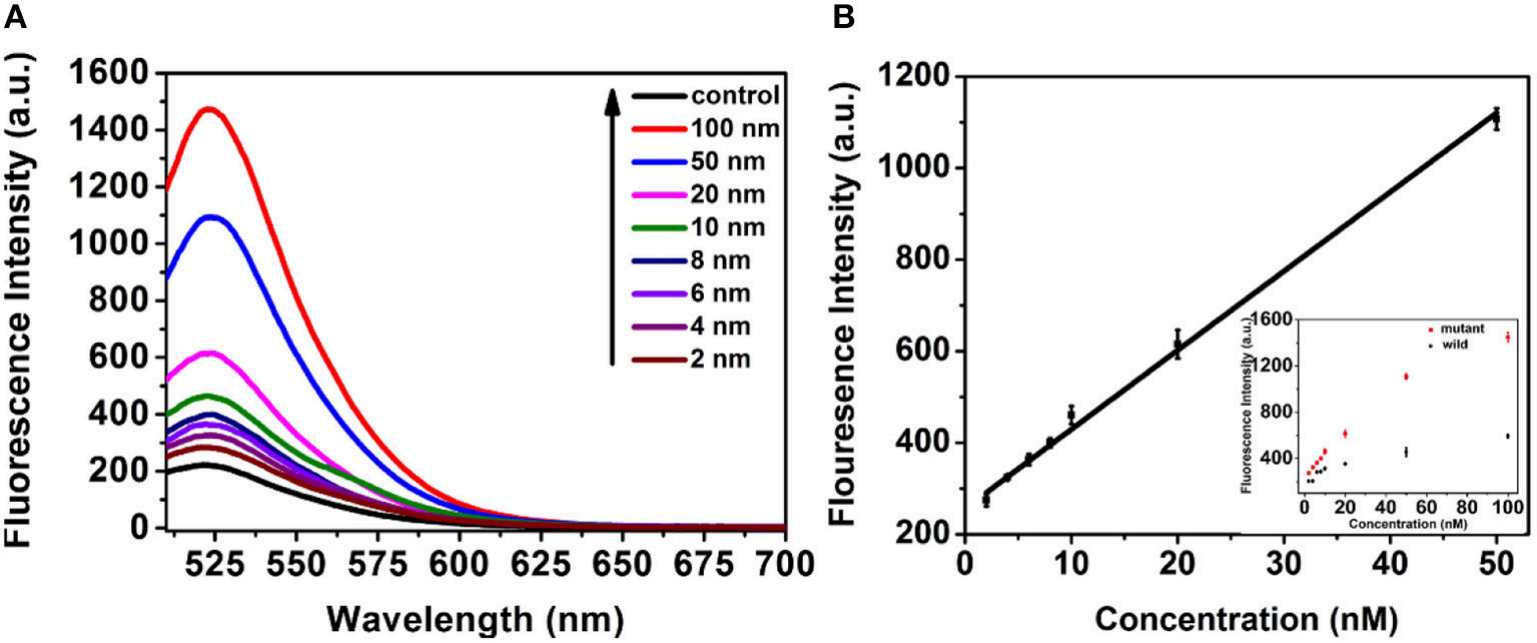
**(A)** Fluorescence response of the assay to control (PBS pH 7.4, 10 mM) and mutant-type ctDNA with different concentrations. **(B)** The Linear relationship between the fluorescence intensity and concentration of mutant-type ctDNA concentration.

Under the optimized conditions, the system was very specific that just produced fluorescence signal toward the mutant-type ctDNA, while no fluorescence signal change was observed even if the concentration of wild-type ctDNA high to 50 nM due to the TSDR probe. Furthermore, the selectivity of the method was detected by using other DNA strands with different sequences. As shown in Figure S2, the fluorescent intensity of mutant-type ctDNA was much higher than that produced by other DNA strands, indicating the great selectivity of the detection strategy. Motivated by the intriguing specificity, the sensitivity of the assay was further investigated for mutant-type ctDNA detection. As shown in Figure 5A, the fluorescence intensity increased with the increasing mutant ctDNA target concentration, and a good linear relationship between the fluorescence intensity and target concentration ranging from 2 to 50 nM was obtained (Figure 5B). The limit of detection (LOD) was calculated to be 0.85 nM using three times of the standard deviation of the control (Wang et al., 2009). To evaluate the practical application, the serum was chosen as the biological sample to evaluate the performance of the detection system. As demonstrated in Figure S3, the mutant ctDNA targets were detected in serum sample solution, implying matrix effects are not a major problem on the reaction system, which indicated the promising potential for clinical application.

## Conclusions

In conclusion, we develop a ctDNA specifical detection method using a pair of DNA clutch separation probes and an ideal discrimination probes based on TSDR. The clutch probes with one of which modified on the MNPs are used to hybridize the mutant ssDNA strand denatured from ctDNA, prevent it reassociation, and separate it in magnetic field to make the other ssDNA strand accessible for hybridization. The discrimination probes were carefully designed and very specific for mutant-type ctDNA, which could efficiently and specifically recognize the mutant-type ctDNA target whereas showed no response to the wild-type ctDNA. Under optimal conditions, the proposed assay displayed a good linear rang and low LOD for target mutant-type ctDNA detection, which was expected to be useful for ctDNA biomedical analysis and clinic theranostic.

## Author contributions

HC designed the experiments and performed the optimization of discrimination probes, ctDNA detection, and wrote the manuscript. YZ and FY performed the characterization of carboxylated MNPs and DNA functionalization of MNPs. CW and HD contributed in project design and manuscript preparation. All authors reviewed the manuscript and approved for submission.

### Conflict of interest statement

The authors declare that the research was conducted in the absence of any commercial or financial relationships that could be construed as a potential conflict of interest.
