# Supplementary material for: ctDNA Detection Based on DNA Clutch Probes and Strand Exchange Mechanism
Source: Front Chem. 2018 Oct 31;6:530. doi: 10.3389/fchem.2018.00530 (PMC6220571; doi:10.3389/fchem.2018.00530)
Supplement: Supplementary file 1 [file Table_1.docx]

**Supporting information**

**ctDNA Detection** **Based on DNA Clutch Probes and Strand Exchange Mechanism**

**Huan Chang^1,2^, Yiyi Zhang^2^, Fan Yang^2^, Changtao Wang^1^*, Haifeng Dong^1,2^***

*^1^Beijing Advanced Innovation Center for Food Nutrition and Human Health, Beijing Technology and Business University (BTBU), Beijing, China, ^2^Beijing Key Laboratory for Bioengineering and Sensing Technology, Research Center for Bioengineering and Sensing Technology, School of Chemistry and Bioengineering, University of Science & Technology Beijing, Beijing, China*


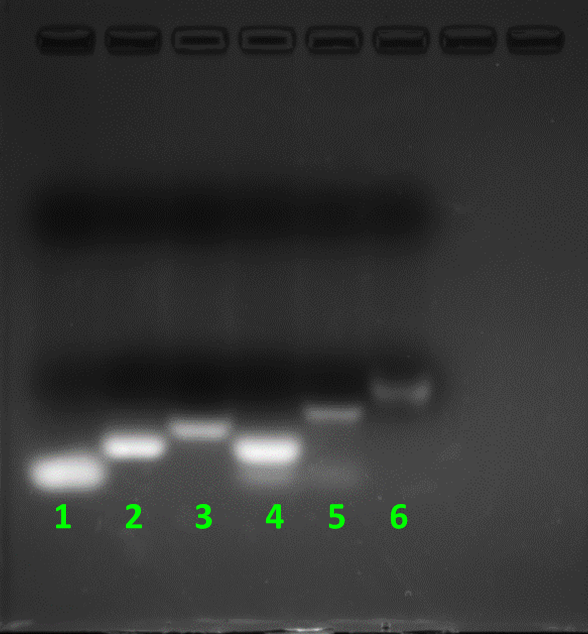


**FIGURE S1.** Electrophoretic analysis of 1) complementary sequences (16 bp), 2) clutch probe 3 (35 bp), 3) clutch probe 5 (44 bp), 4) clutch probe 3 and clutch probe 5, 5) complementary sequence hybridize with clutch probe 3 and 6) complementary sequence hybridize with clutch probe 3 and clutch probe 5.





**FIGURE S2.** PL spectra of the solution to verify the selectivity of the assay over several DNA strands.


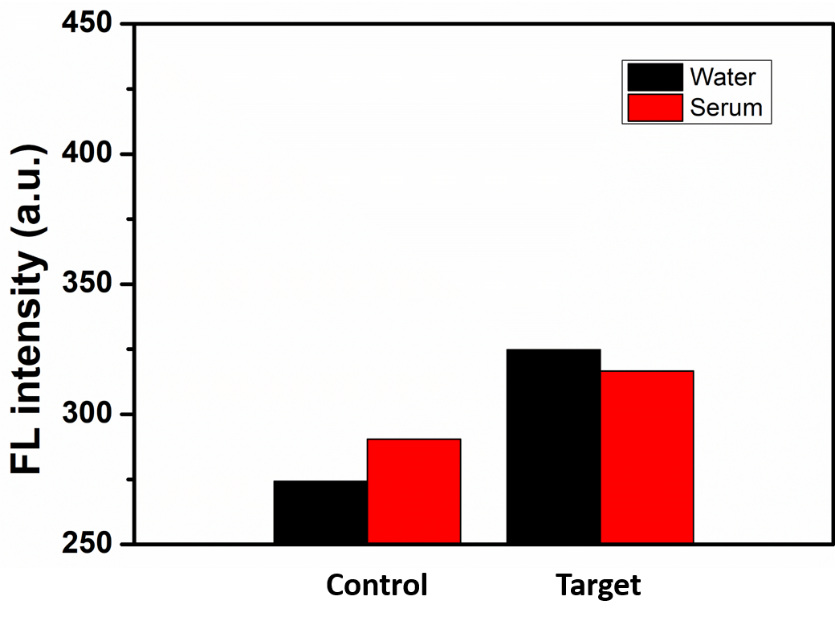


**FIGURE S3.** PL intensity of the solution without or with target DNA strands.
